# A multifunctional optoelectronic device based on 2D material with wide bandgap

**DOI:** 10.1038/s41377-023-01327-8

**Published:** 2023-11-22

**Authors:** Hongwei Xu, Jingwei Liu, Sheng Wei, Jie Luo, Rui Gong, Siyuan Tian, Yiqi Yang, Yukun Lei, Xinman Chen, Jiahong Wang, Gaokuo Zhong, Yongbing Tang, Feng Wang, Hui-Ming Cheng, Baofu Ding

**Affiliations:** 1 Faculty of Materials Science and Engineering/Institute of Technology for Carbon Neutrality, Shenzhen Institute of Advanced Technology, Chinese Academy of Sciences, Shenzhen, Guangdong 518055 China; 2 School of Semiconductor Science and Technology, South China Normal University, Foshan, Guangdong 528225 China; 3 Shenzhen Institute of Advanced Technology, Chinese Academy of Sciences, Shenzhen, Guangdong 518055 China; 4 Hubei Three Gorges Laboratory, Yichang, Hubei 443007 China; 5 Shenzhen Geim Graphene Center (SGC), Tsinghua-Berkeley Shenzhen Institute (TBSI) & Tsinghua Shenzhen International Graduate School (SIGS), Tsinghua University, Shenzhen, Guangdong 518055 China

**Keywords:** Liquid crystals, Photonic devices

## Abstract

Low-dimensional materials exhibit unique quantum confinement effects and morphologies as a result of their nanoscale size in one or more dimensions, making them exhibit distinctive physical properties compared to bulk counterparts. Among all low-dimensional materials, due to their atomic level thickness, two-dimensional materials possess extremely large shape anisotropy and consequently are speculated to have large optically anisotropic absorption. In this work, we demonstrate an optoelectronic device based on the combination of two-dimensional material and carbon dot with wide bandgap. High-efficient luminescence of carbon dot and extremely large shape anisotropy (>1500) of two-dimensional material with the wide bandgap of >4 eV cooperatively endow the optoelectronic device with multi-functions of optically anisotropic blue-light emission, visible light modulation, wavelength-dependent ultraviolet-light detection as well as blue fluorescent film assemble. This research opens new avenues for constructing multi-function-integrated optoelectronic devices via the combination of nanomaterials with different dimensions.

## Abstract (web summary)

A new type of luminescent nanocomposite based on 0D/2D vertical heterojunction is prepared, enabling the fabrication of a multifunctional optical device for blue light emission, light modulation and UV detection.

## Graphical abstract

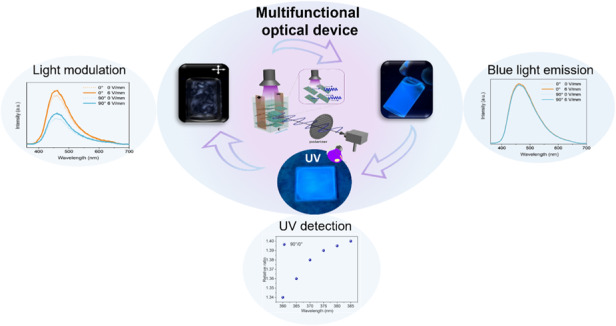

## Introduction

Low-dimensional materials usually have size-dependent quantum confinement effects due to the nanoconfinement in one or more dimensions^1–3^. Taking two-dimensional (2D) material as an example, with thickness reduced to a single atomic layer, its geometric anisotropy is larger than that of other dimensional materials by two to three orders of magnitude^4–7^. Recent research reveals a close relationship between geometric anisotropy and optical anisotropy. Alignment of geometrically anisotropic 2D materials can endow the resultant dispersion with extremely large optical anisotropy, allowing for many exceptional potentials in diverse applications. For instance, they can be utilized in the assembly of special functional materials, unconventional optical modulation, and detection, such as constructing optical hydrogels, enabling deep ultraviolet (UV) modulation, and facilitating visual ion chemical sensing^8–12^.

In the meantime, another important low-dimensional material, quantum dot, has all three dimensions in the nanoscale, exhibiting enhanced quantum confinement effects, which leads to the opening of band gap^13–15^. The band gap is quantitatively dependent on the size in all three dimensions. By synthesizing quantum dot materials of different sizes, it becomes feasible to achieve continuously tunable band gaps, thereby offering potential for diverse colored light emission, especially for blue light emission. For example, perovskite dots, and carbon dots as representatives of quantum dots, demonstrate the advantages of high color purity, wide color gamut, and continuously tunable emission peak wavelength^16–19^. However, quantum dots possess a relatively uniform size in all three directions, resulting in a light emission with optical isotropy.

Recent research has focused on the combination of low-dimensional materials, encompassing not only the composite of same-dimensional materials but also the construction of other different low-dimensional materials^20–24^. Combination of different low-dimensional materials with complementary properties had led to performance breakthroughs in the various application fields. For instance, the combination of quantum dots of Fe_2_O_3_/2D C_3_N_4_ materials as well as CsPbBr_3_ quantum dots/Bi_2_WO_6_ nanosheets demonstrated an improvement in Fenton catalytic performance or photocatalytic CO_2_ reduction^25,26^. Similarly, the combination of nanorod of Co_3_O_4_ and 2D graphene materials led to enhanced key parameters of supercapacitor devices^27^, while the compound of quantum dot Ag_3_PO_4_ and nanorod of TiO_2_ materials resulted in efficiency-improved photodegradation^18,28^. Hence, the composite of materials in different dimensions have emerged as a burgeoning interdisciplinary field^29^.

In this work, we demonstrate the further application extension of the nanocomposite in the construction of a multifunctional optoelectronic device, which comprises carbon blue quantum dots and 2D cobalt-doped titanium oxide. Such a device is capable of emitting and modulating blue light as well as modulating and detecting UV light. The nanocomposite in the dispersion is aligned by an external field, consequently serving as a tool to assemble the highly-ordered fluorescent film with uniform blue-light emission. This combination based on wide-bandgap nanosheets and quantum dots enables the fabrication of a multifunctional optoelectronic device. The device may open up new possibilities for future high-performance optical devices and related optical applications, such as polarization-tunable light source in a polarizer-free manner, self-modulated light emission, polarization-based data communication, and ultraviolet-based optical detection^30,31^.

## Results

**Fig. 1:**
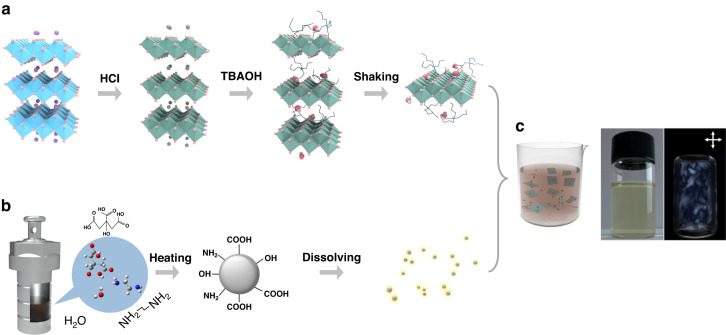
Preparation of carbon dots/2D material composite solution. Three-step exfoliation method for preparing monolayer 2D cobalt-doped titanium oxide (**a**). One-step hydrothermal method for preparing carbon dots (**b**). The formation of carbon dots/2D material luminescent nanocomposite (**c**)

We synthesized 2D material, as depicted in the Fig. 1a^32,33^. We started with uniformly blending of the raw materials in the given ratio, followed by high-temperature recrystallization, yielding a layered bulk material with potassium ions predominantly interspersed between the layers, while lithium-doped titanium oxide mainly dominates on both flanks. Subsequently, via a series of controlled processes, such as mild acid soaking and stirring, we engendered an ion exchange between hydrogen ions and potassium ions, as well as partial lithium ions within the bulk titanium oxide structure. This ion exchange engenders two significant outcomes: first, an expansion of the interlayer spacing and second, the facile ionization of hydrogen ions within the titanium oxide structure in the aqueous solution, resulting in surface charge accumulation on the titanium oxide layers. Consecutively, by introducing organic bases and engaging in acid-base neutralization, larger-sized tetrabutylammonium ions were judiciously inserted into the interlayers, effectively displacing the hydrogen ions. This further expansion of the interlayer spacing renders the layered material highly amenable to exfoliating into monolayers through a gentle and soft mechanical vibration. Subsequently, following a period of controlled vibration or gentle agitation, the material readily culminated in the successful isolation of single layers.

The carbon-dot solution was synthesized on the basis of previous work^34,35^. Generally, as schemed in Fig. 1b, ethylendiamine (336 μL) and citric acid (1.05 g) were mixed and stirred into deionized water (10 mL). Subsequently, the well-dissolved solution was poured into a Teflon-lined autoclave and the solution was maintained at 150 °C (5 h) for sufficient reaction. Finally, the prepared samples were naturally cooled down to room temperature. Before characterization, the dialyzed product was treated by the following steps: absorb 1 mL original solution and dilute 300 times in deionized water. Accordingly, the color of dispersion solution changed from yellowish to colorless after the dilution. Upon obtaining stable aqueous dispersions of both carbon dots and 2D cobalt-doped titanium oxide materials, we embarked on a meticulous coalescence process, ensuring a uniform mixture. The tested negative electronegativity indicates the negatively charged surface of the 2D material. The carbon dot was judiciously electro-adsorbed onto the outer surface of nanoscale material, effectuating a nanocomposite linkage between the carbon dots and the 2D carrier. Figure 1c shows a schematic diagram of the nanocomposite solution, the corresponding solution photographs with and without the crossed polarizers (from left to right). Figure S1 shows an obvious Tyndall phenomenon under 635 nm laser irradiation, indicating that the solution is a colloidal one. When colloidal suspension is shaken, blue birefringent pattern is displayed in the presence of crossed polarizers (seen in the right of Fig. 1c), which is attributed to the flow-induced birefringence effect and photoluminescence effect of the nanocomposite.

**Fig. 2:**
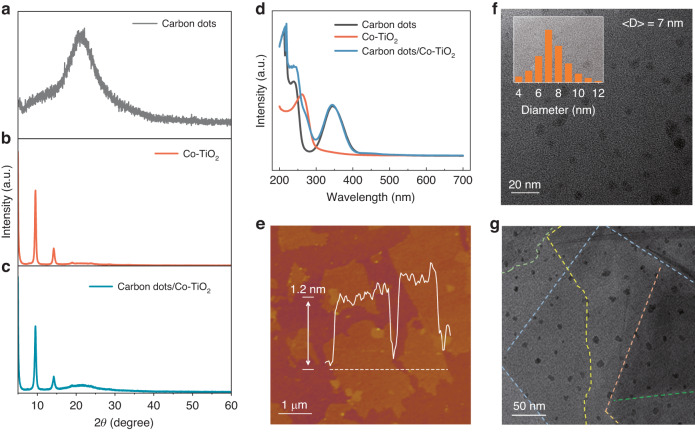
Characterization of carbon dots, 2D cobalt-doped titanium dioxide nanosheets and their nanocomposite. XRD characterization of carbon dots (**a**), 2D cobalt doped titanium dioxide (Co-TiO_2_) nanosheets (**b**) and their nanocomposite (**c**). UV–Vis absorption spectrum of all three material systems (**d**). AFM characterization of 2D cobalt doped titanium oxide (**e**). TEM images of dispersed carbon dots (**f**) and their nanocomposite (**g**)

Figure S2 demonstrates the synthesized samples are composed of Ti, O, and Co elements, and these elements are uniformly distributed in the 2D nanosheet. Moreover, to provide additional evidence, we performed an XPS analysis, revealing two characteristic Co 2p peaks at 779.7 eV and 795.5 eV, corresponding to the typical binding energies of Co 2p_3/2_ and Co 2p_1/2_, respectively (Fig. S3). These results collectively verify the successful and uniform dopant of cobalt in the titanium oxide nanosheets. Figure 2a–c shows the XRD patterns of both the as-synthesized carbon dot, 2D cobalt-doped titanium oxide and their composite samples, respectively. In Fig. 2a, the XRD pattern of carbon dots displays a broad diffraction peak approximately at 2*θ* = 23°, corresponding to the (002) crystal plane spacing of carbon dots. The XRD spectra of the prepared cobalt-doped titanium oxide nanosheets are shown in Fig. 2b. In the range of 2*θ* = 5° ~ 30°, each major diffraction peak is consistent with that of the reported single-layer 2D cobalt-doped titanium oxide^36,37^.

When compared to the undoped sample, only a minor shift of the (020) peak towards lower angles is observed in the cobalt-doped sample (Fig. S4). This observation suggests that larger Co cations have effectively replaced Ti cations sites within the 2D titanium oxide nanosheet, while the crystalline structure is well maintained. The 2D material can be stably dispersed in an aqueous solution and is convenient for subsequent recombination with carbon dots. As displayed in Fig. 2c, it can be seen apparently that the XRD data of the combined system of carbon dots and 2D materials can be well accorded with the above-mentioned analysis results. Moreover, Fig. 2d shows the absorption spectrum of carbon dot, 2D material and their composite system. It can be seen clearly that the absorption range of carbon dots covers all UV region and instead, the response range of 2D material is limited to 300 nm, which is consistent with the reported bandgap of about 4.0 eV for 2D cobalt-doped titania^20,33^. It elaborates that the existence of cobalt-doped titania nanosheets will not affect the luminescence properties of carbon dots. Figure 2e and S5 show the single-layer atomic force microscopy (AFM) picture and the stacked scanning electron microscopy (SEM) picture of 2D material prepared by soft chemical exfoliation method, respectively. The AFM image reveals that a typical 2D cobalt-doped titanium oxide nanosheet exhibits a lateral size of 2 μm and a thickness of 1.2 nm (Fig. 2e). Detailed statistical results regarding the lateral size and thickness are provided in Fig. S6, demonstrating an average lateral size of about 1.8 μm and an average thickness of about 1.17 nm, respectively. Such results give a consequent geometric anisotropy ratio of 1500 (lateral size/thickness). TEM test on carbon dots is conducted, as shown in Fig. 2f. The inset of Fig. 2f illustrates the particle size distribution of 0D carbon dots, counted from TEM image. Through fitting, the average diameter of the carbon quantum dots was determined to be ~7.2 nm. TEM image (Fig. 2g) shows that 0D carbon dots are loaded on the surface of 2D cobalt-doped titanium oxide nanosheets. No aggregation among carbon dots occurred, indicating their homogeneous distribution when contacting with the nanosheets, which is the base for the formation of nanocomposite of 0D carbon dots and cobalt-doped titanium oxide nanosheet.

**Fig. 3:**
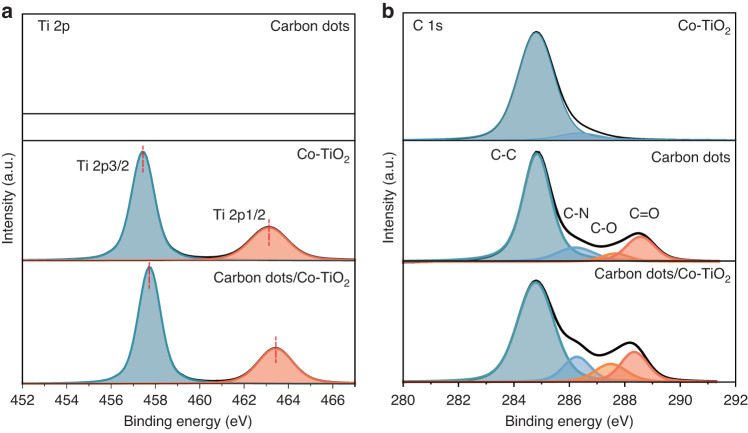
FT-IR and XPS analysis of carbon dots/2D cobalt doped titanium dioxide nanosheets. The deconvoluted XPS spectra of Ti 2p (**a**) and C 1s (**b**) in carbon dots, 2D cobalt-doped titanium dioxide nanosheets as well as their nanocomposites

It is worth mentioning that in Fig. S7, the zeta potential of the synthesized nanocomposite system is about −22.7 mV, which is similar to that of known cobalt-doped titania material (<−30 mV)^38,39^. Electrostatic absorption may attract light-weighted carbon dot to adhere on 2D material, resulting in forming nanocomposite. To further judge interface contact of carbon dots/2D cobalt-doped titanium oxide heterostructure, the testing and analysis of X-ray photoelectron spectroscopy (XPS) and Fourier transform infrared spectroscopy (FT-IR) were performed successively. The full survey spectrum (Fig. S8) of the carbon dots/2D cobalt-doped titanium oxide composites show some peaks corresponding to titanium (Ti 2p), cobalt (Co 2p), carbon (C 1s), nitrogen (N 1s), and oxygen (O 1s). Notably, no Ti-C bond peak was observed in the Ti 2p spectra of the nanocomposite (Fig. 3a) or in the C 1s spectra of the carbon dots (Fig. 3b), but cobalt-doped titanium oxide nanosheets show a shift toward high bond energy after loading carbon dots. This observation confirms no apparent interaction between Ti and C in carbon dot when being anchored with the carbon dots, only inducing the increase of Ti 2p valence state. Furthermore, distinct C-O (287.2 eV) vibrational peak is prominently evident in the C 1s (Fig. 3b) within the carbon dots/2D cobalt-doped titanium oxide nanocomposite. The higher intensity of this peak is in sharp contrast to the zero intensity of cobalt-doped titanium oxide and the low intensity of carbon dots, indicating C-O bond increase. The above results of the absence of Ti-C bond, the lifting of Ti 2p valence state, and the increase of C-O bond, collectively indicate that the carbon dots are loaded onto the 2D nanosheets through the formation of Ti-O-C bonds, induced by chemical adsorption^40,41^. The formation of the carbon dots/2D cobalt-doped titanium oxide composites is further verified through the analysis of FT-IR spectra, as depicted in Fig. S9. Hydroxyl (C-OH, 3546 cm^−1^) and carboxyl (O=C-O, 1713 cm^−1^) of carbon dots could react with the hydroxyl (TiO_2_-OH, 3420 cm^−1^) at the surface of TiO_2_ to form Ti-O-C bonds. FT-IR spectra of cobalt-doped titanium oxide, the carbon dots/2D cobalt-doped titanium oxide nanocomposite, and carbon dots are presented in Fig. S9a. C-OH and O=C-O are shown in the FT-IR spectrum of pure carbon dots, and TiO_2_-OH is displayed in the FT-IR spectrum from cobalt-doped titanium nanosheets, which offer a sufficient condition for the formation of Ti-O-C bond. All the prominent absorption peaks of carbon dots and cobalt-doped titanium oxide nanosheets are distinctly evident in the FT-IR spectra of the carbon dots/2D cobalt-doped titanium oxide nanocomposites. Notably, an enhanced absorption peak emerges at 1393 cm^−1^ for the composite, which is dominant from the formation of C-O bonds. In contrast to cobalt-doped titanium oxide, the composite shows an overall broadening of the Ti-O bond peaks in the 400–600 cm^−1^ range, which can be attributed to a combination of Ti-O-Ti and Ti-O-C vibrations. (Fig. S9b)^42,43^. We note that to form the uniformly dissolved nanocomposite, the concentration should be in the proper range as higher concentration (>0.3 vol‰) carbon dots/2D cobalt-doped titania composites tend to aggregate (Fig. S10).

**Fig. 4:**
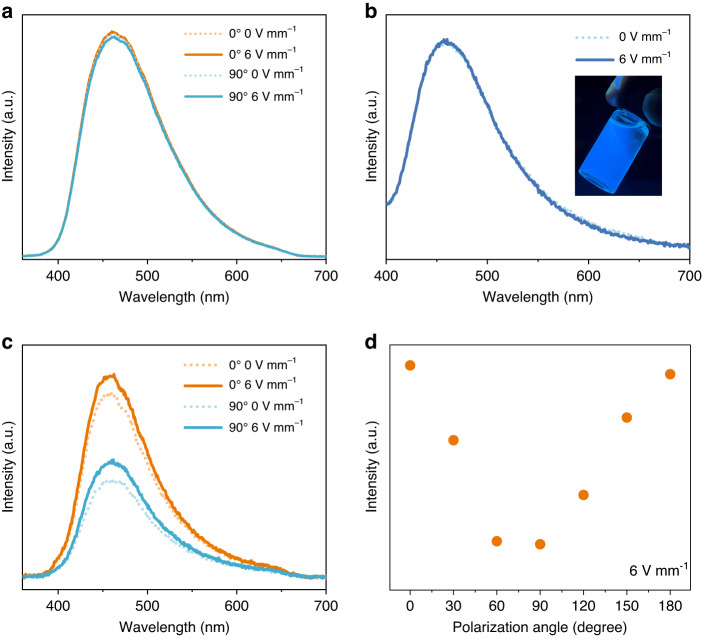
Optical anisotropy of emitted light from nanocomposite of carbon dot/2D material. Dependence of light vibration from carbon dot (**a**) or carbon dot/2D material (**b**, **c**) on the electric field and transmission angle of the analyzer (**d**). Inset shows the blue light emission from the nanocomposite of carbon dot/2D material under 365 nm UV irradiation. The analyzer is absent (**b**) and present (**c**) on the optical path

This meticulously engineered microscale carbon dot/2D nanoscale heterostructure seamlessly combines the distinctive attributes of carbon dots with those of the 2D carrier enabling the design of an optoelectronic device with tunable light emission and detection. We consequently construct a device that includes the excited light source, dispersion of composite-dimensional nanomaterial, and electrodes with their electric field perpendicular to the optical path. Under the influence of an external electric field, the 2D material serves as a microscopic motor, carrying the carbon dot materials, inducing flipping and alignment congruent with the external field. Usually, the carbon dot is geometrically isotropic. Therefore, it can be speculated that the solution with carbon dots will emit light independent of the emission angle, namely, optically isotropic light. In other words, even driven by the electric field, the emitted light shows independence from the electric field. This is consistent with the result in Fig. 4a. The photoluminescent spectrum from the carbon-dot-only device keeps identical when rotating the analyzer from 0^o^ to 90^o^, relevant to the electric flux.

Nonetheless, for a single nanocomposite of carbon dot/2D material, due to its geometrical anisotropy, which may lead to the vibration-angle-dependent intensity of light emitted from the absorbed carbon dot. To quantitatively assess this anisotropic light emission, we devised an optoelectronic device coupled with an analyzer to evaluate the ratio of light intensities in two orthogonal vibration directions: parallel and perpendicular to the electric field. The cuvette is loaded with nanocomposite of carbon dot/2D material at the concentration of 0.01 vol‰, which is set for precluding exciton quenching effects engendered by excessive concentrations in the solution and suppressing the scattering effects. Owing to the location of the electrodes outside the optical path, the total area and effective nanocomposite thickness of the blue light emitting device in the optical path reach 4 cm^2^ and 25 nm, accounting for the final high visible transparency of 75% (Fig. S11). The state-of-the-art electro-optic device shows the blue light emission from carbon dots at the peak wavelength of 460 nm.

As shown in Fig. 4b, without an analyzer, the spectrum remains the same regardless of the exertion of electric field. Once an analyzer is placed on the optical path, the intensity of emission spectrum shows a close relationship with angle of the transmission axis of analyzer relevant to the electric field (Fig. 4c). The emission light is stronger in the case that the transmission axis of analyzer is parallel to the electric flux than the vertical counterpart (Fig. 4d). Figure S12 shows the schematic diagram of the dimming experiment principle of the fabricated device. When the electric field is applied, the alignment of the 2D material will drive the directional arrangement of the loaded carbon dots, thereby maximizing the luminescence intensity in a specific direction for obtaining enhanced polarized luminescence. It is worth mentioning that the composite system does not affect the optical properties of carbon dots (see Fig. S13). To quantitatively describe the impact of electric field on the anisotropic emission, we defined the current efficiency *E*_*c*_ by dividing the anisotropic luminance differences *ΔB* with driving current density *I*, *E*_*c*_ = *ΔB/I*. The luminance difference *ΔB* of 150 cd m^−2^ is measured at the current density *I* of 1 mA cm^−2^, giving rise to the current efficiency *E*_*c*_ of 15 cd A^−1^. Such large anisotropic current efficiency is consequently ascribed to both the alignment and extremely large optical anisotropy of 2D material for the isotropic light emitted from carbon dots.

**Fig. 5:**
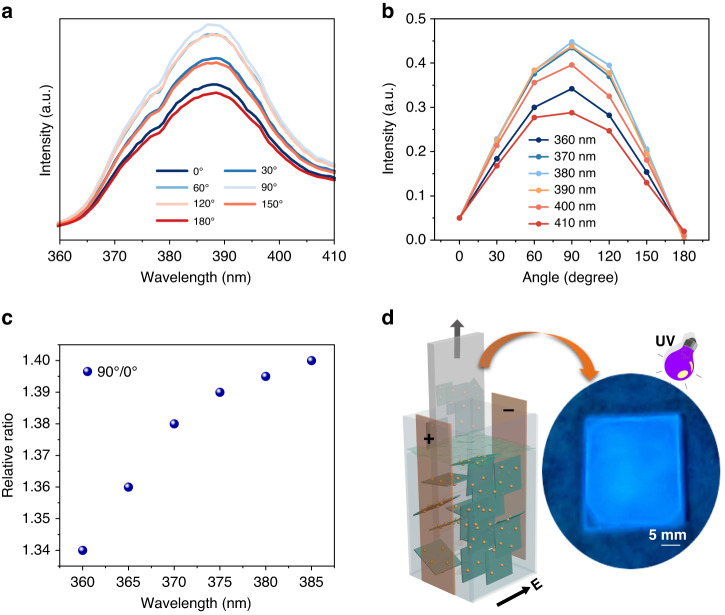
Anisotropic transmittance of UV light for the electro-optic device based on the composite of carbon dot and 2D material. Transmittance spectrum versus angle between the transmittance axis of the analyzer and electric field (**a**). Relative transmittance intensity of UV light versus polarizer rotation angle with six selected wavelengths (**b**). Linear dependence between the relative ratio of transmittances at polarizer angle of 90^o^ and 0^o^ with wavelengths (**c**). Preparation of inch-scale nanocomposite film of carbon dot and 2D material via pulling the flexible substrate from electro-optic device (**d**)

By finely tuning the concentration, we further engineer the transmittance within the UV spectrum. By decreasing the concentration to 0.05 vol‰, we manifest an average transmittance exceeding 65%. The effective thickness of the device in the optical path is about 38 nm. The device can function as vertical heterostructure-based UV detector. Exerting an external electric field, we observed the correspondence of UV transmittance with the angle between transmission axis and external electric flux in the spectral range of 360 to 410 nm (Fig. 5a). Similar to the above analysis, the angle-dependent UV transmittance is possibly from the anisotropic absorption by aligned nanocomposite. In detail, the electrical field can re-orient the nanocomposite. This alignment of 2D material, together with the UV light absorption of carbon dots endow the electro-optic device with the function of anisotropic UV absorption. To quantify the anisotropic UV absorption, we plot the correspondences of intensity with the angle for six specific wavelengths in the UV spectral range (Fig. 5b). The maxima of absolute value occur at about 90^o^, indicating the minimum UV absorption in the vertical vibration direction relevant to the electric field. More importantly, such anisotropic UV transmittance is wavelength dependent. The substantiation of this feature is notably evident through the observable deep UV anisotropic absorption. Exploiting the well-established relationship between dichromic absorption and wavelength, we glean valuable insights into the optical anisotropy ratio at different wavelength of UV light by subjecting the device to external field conditions.

Figure 5c shows relative ratios between light intensities at polarization angle of 90° and 0°, which has a linear dependence on the wavelength of UV light, thereby effectively realizing deep UV light detection functionality. Finally, based on the ordered arrangement of the low dimensional nanocomposite in the electro-optic device under an external electric field, we used a conductive flexible ITO electrode as the substrate and achieved the preparation of highly ordered multilayered nanocomposite thin film of carbon dot and 2D material at the wafer scale (Fig. 5d). Briefly, the film is fabricated by pulling the flexible substrate using the Langmuir-Blodgett (LB)-like method under the action of an external electric field. The as-prepared nanocomposite device can serve as a flexible vertical heterojunction optical device with blue light emission, and has an average thickness of <25 nm (corresponding to about 20 layers of 2D material), and exhibits transparency of >75% without excited light or uniform blue light emission throughout the entire film area under excited UV light. Such device can be applicable as the backlight of display, surface light source of lighting and flexible UV detector.

## Discussion

In summary, we have demonstrated an in-situ linking approach, enabling the creation of a nanoscale heterostructure by combining blue-emissive carbon dot with highly electrically sensitive cobalt-doped titanium oxide in an aqueous dispersion. This heterostructure seamlessly integrates the distinctive functionalities of both materials, encompassing highly efficient blue light emission and remarkable sensitivity to external fields. Additionally, it demonstrates the modulation capability of blue and UV light, as well as UV light detection, thereby offering a trifecta of functionalities. Our strategy effectively addresses the concern of combining different dimensional material thereby achieving the unique and multiple optoelectronic properties that are unattainable in conventional pure low-dimensional material.

## Materials and methods

All chemical reagents were purchased from Alfa Aesar or Macklin and used without further purification. The cobalt-doped titania powder was synthesized following our previous reported work^20^. All measurements reported in this work were made at a temperature of 25 °C. X-ray diffraction (XRD) measurement was performed on the Advanced XRD diffractometer equipped with Cu-Kα monochromator (*λ* = 1.5418 Å) at a scanning rate of 10°/min from 10° to 70° (Bruker D8, Germany). The fluorescence spectra in the aqueous suspension were recorded by using a fiber optic spectrophotometer with NOVA-HL200 (Ideaoptics, shanghai). UV–Vis reflectance spectra were collected by a Cary 5000 spectrophotometer (Agilent, USA). The morphology of the intermediate products was examined using AFM (Cypher ES, UK) and Hitachi SU8010 scanning electron microscopy (SEM) at 5 keV (Oxford Instruments, UK). Zetasizer Nano ZS was capable of zeta-potential measurement fitted with a high-concentration zeta-potential cell (Malvern Instruments, UK). Transmission electron microscopy (TEM) (200 kV, F200X Talos, FEI, USA) was used to characterize morphology of exfoliated cobalt-doped titanium oxide nanosheets and Energy Dispersive X-Ray Spectroscopy was used to analyze elemental distribution. XPS analysis was performed for elemental analysis using the 5000 versa probe III (Ulvac-Phi, Japan) and FT-IR spectra were conducted to analysis the composition of compounds (Agilent Cary 660, USA).

Electro-optical measurements: In the main text, a 365 nm UV LED was used as the incident light source. The colloid sample (suspension of mixed 2D cobalt doped titania and carbon dots in 0.5 cm × 1.0 cm cross-section and 5.5 cm tall quartz cuvette) was placed between two crossed polarizers (extinction ratio 10^5^:1, Glan-Laser Calcite Polarizers, GL10-A, Thorlabs Inc.), with an electric field supplied by a home-made copper electrode applied in a direction perpendicular to the optical path and at 45° to the polarizer/analyzer. The electric field was controlled using an oscilloscope, signal generator, and amplifier. Transmitted light was detected by a PM100A (Thorlabs Co, USA).

### Supplementary information

Supporting information for A multifunctional optoelectronic device based on 2D material with wide bandgap

## Data Availability

The authors declare that all data supporting the results reported in this study are available within the paper and the Supplementary Information. Additional data used for the study are available from the corresponding author upon reasonable request.
