# Supplementary material for: A multifunctional optoelectronic device based on 2D material with wide bandgap
Source: Light Sci Appl. 2023 Nov 22;12:278. doi: 10.1038/s41377-023-01327-8 (PMC10663625; doi:10.1038/s41377-023-01327-8)
Supplement: Supplementary file 1 — Supporting information for A multifunctional optoelectronic device based on 2D material with wide bandgap [file 41377_2023_1327_MOESM1_ESM.docx]

**Supporting information for**

**A multifunctional optoelectronic device based on 2D material with wide bandgap**

Hongwei Xu^1^, Jingwei Liu^1^, Sheng Wei^1^, Jie Luo^1^, Rui Gong^1^, Siyuan Tian^2^, Yiqi Yang^2^, Yukun Lei^2^, Xinman Chen^2^, Jiahong Wang^3,4^, Gaokuo Zhong^3^, Yongbing Tang^1^, Feng Wang^1^***,** Hui-Ming Cheng^1^***** and Baofu Ding^1,5^*****

^1^Faculty of Materials Science and Engineering/Institute of Technology for Carbon Neutrality, Shenzhen Institute of Advanced Technology, Chinese Academy of Sciences, Shenzhen 518055, Guangdong, China

^2^School of Semiconductor Science and Technology, South China Normal University，Foshan

^3^Shenzhen Institute of Advanced Technology, Chinese Academy of Sciences, Shenzhen 518055, Guangdong, China

^4^Hubei Three Gorges Laboratory, Yichang, Hubei 443007, China

^5^Shenzhen Geim Graphene Center (SGC), Tsinghua-Berkeley Shenzhen Institute (TBSI) & Tsinghua Shenzhen International Graduate School (TSIGS), Tsinghua University, Shenzhen 518055, China

Corresponding author: Feng Wang (f.wang@siat.ac.cn), Hui-Ming Cheng (hm.cheng@siat.ac.cn) and Baofu Ding (bf.ding@siat.ac.cn)

**Experimental section of 2D cobalt-doped titanium oxide nanosheets**

The synthesis process of cobalt-doped titanium oxide materials consists of the following three steps: In step 1, layer compound KLiTiCoO*_x_* was prepared by a high-temperature solid reaction method. The Molar ratio of TiO_2_, CoO, K_2_CO_3,_ and Li_2_CO_3_ precursor is 25:3:6:1, and then were mixed grinding in a stoichiometric ratio and calcined at 1100 ℃ for more than 10 h. In step 2, K^+^ and Li^+^ in KLiTiCoO*_x_* were replaced by H^+^ via protonic exchange and formed HTiCoO*_x_* compound. Concretely speaking, 200 mL of diluted hydrochloric acid (1 M) was added into the prepared KLiTiCoO*_x_* powder (1 g) and fully stirred for about 3-4 days to allow sufficient exchange of ions. In step 3, interlayer H^+^ was replaced by tetrabutylammonium (TBA^+^) in the final obtained few-layer TiCoO*_x_* via ionic exchange. To be more specific, the power of HTiCoO*_x_* compound was soaked in TBAOH solution (10 mL) and softly shaken through an oscillating table for 5 hours. The formation of the liquid crystal phase of the nanosheet can be clearly seen by slight shaking and the obtained products are then dispersed into a certain concentration of deionized water by centrifugation for further experiments.


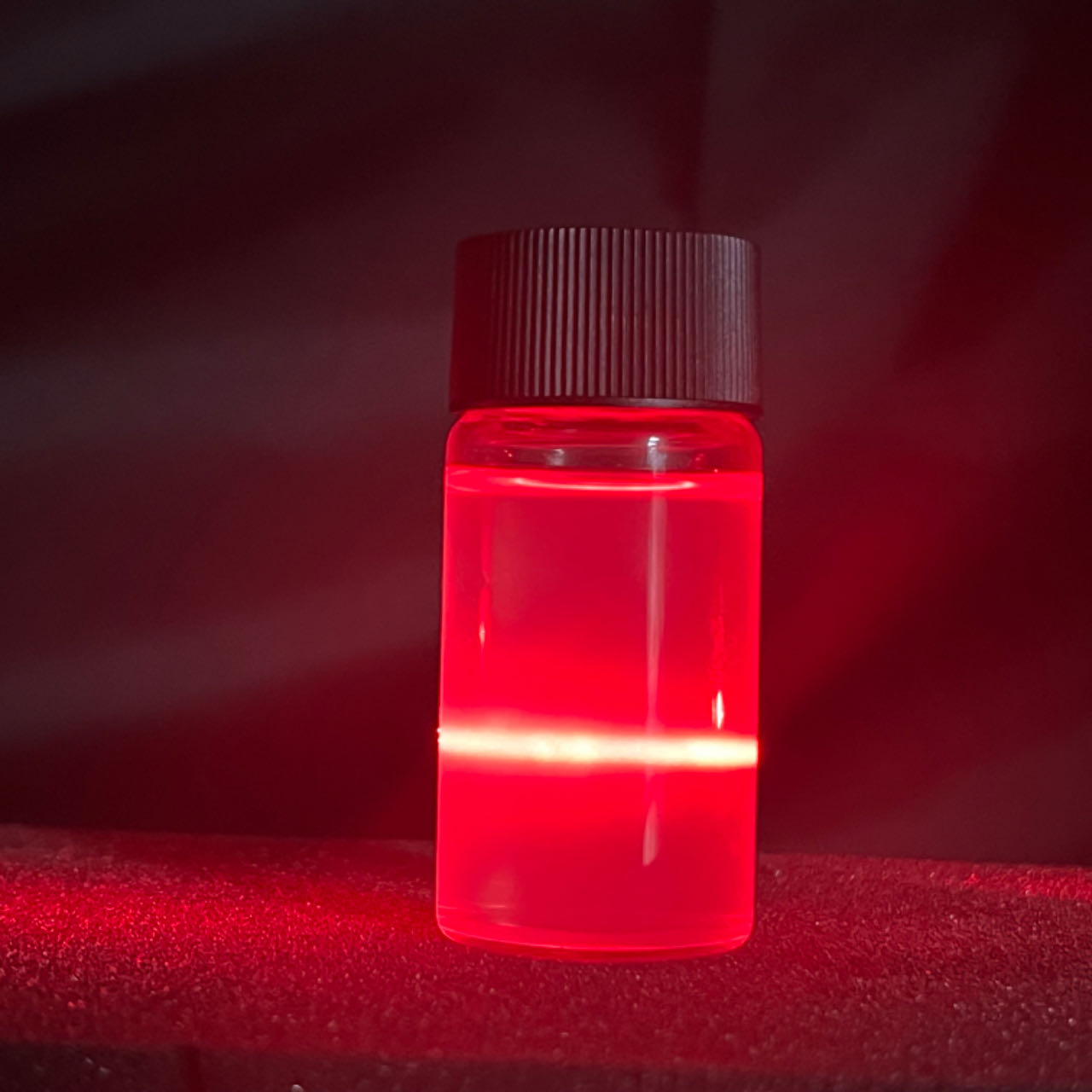


**Fig. S1** The Tyndall phenomenon of hetero-structural solution under 635 nm laser irradiation.


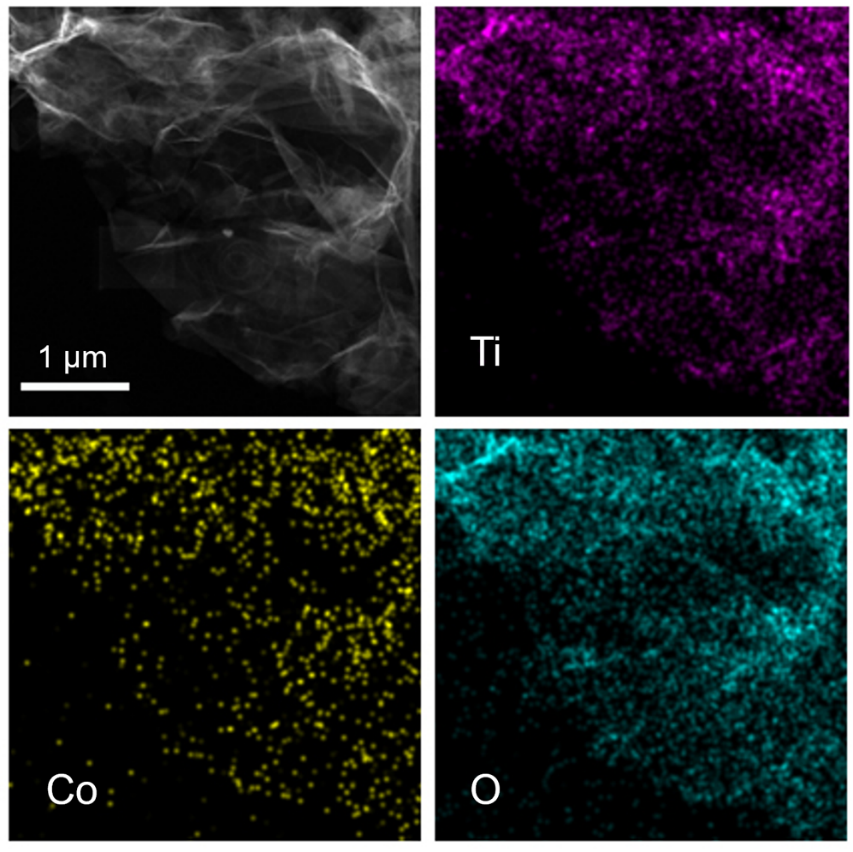


**Fig. S2** EDS mapping of the elemental composition of 2D cobalt-doped titanium oxide nanosheet as well as uniform Co doping with detectable atomic of O, Ti, and Co.


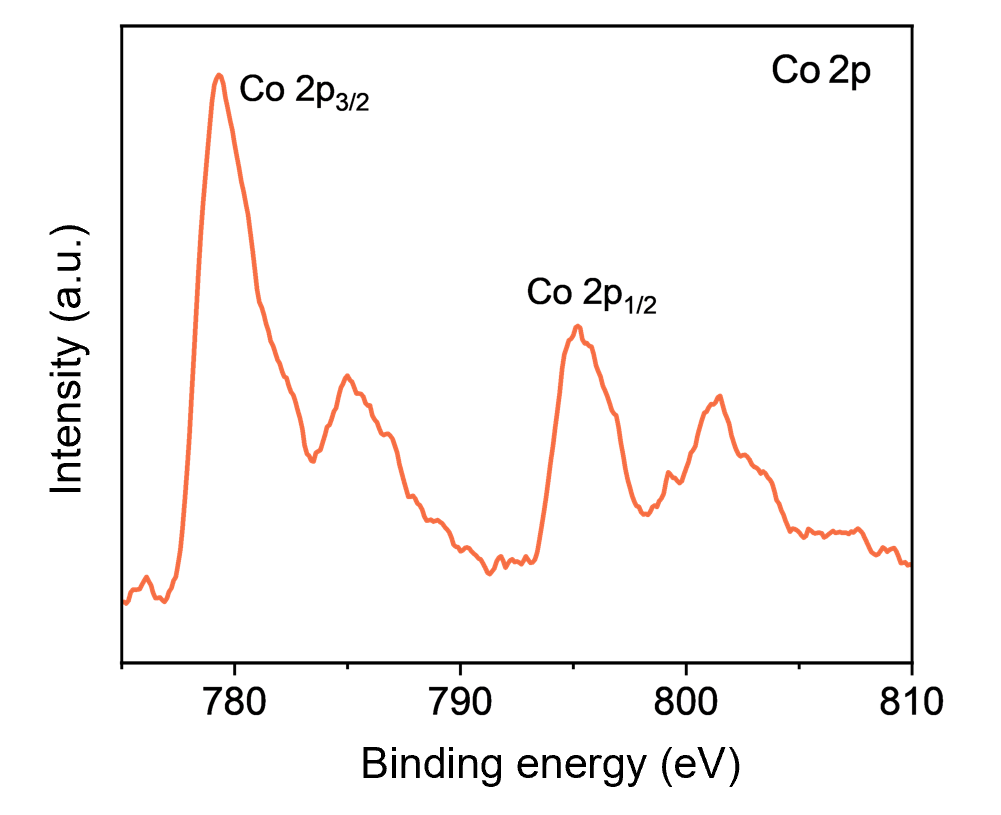


**Fig. S3** The binding energy of Co 2p in 2D cobalt-doped titanium oxide nanosheets.


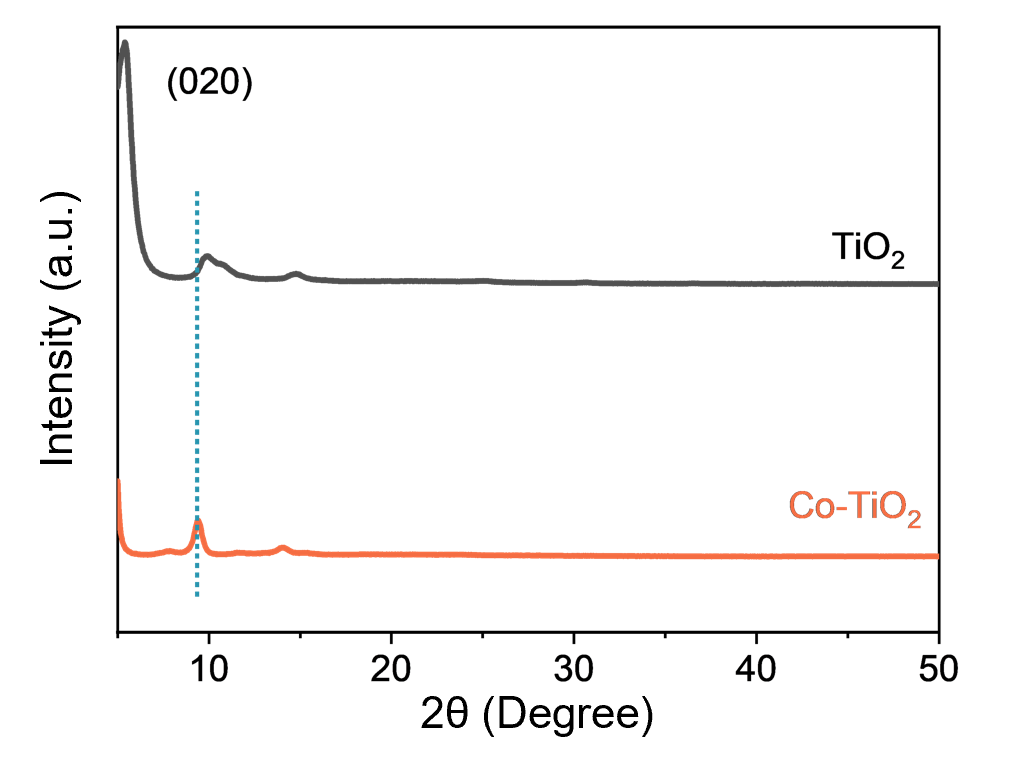


**Fig. S4** XRD characterization of titanium oxide nanosheets and cobalt-doped titanium oxide nanosheets.


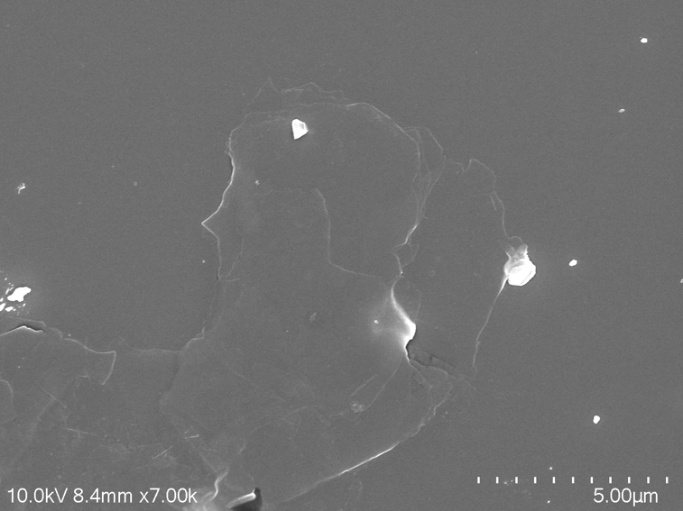


**Fig. S5** The SEM image of the stacked cobalt-doped titanium dioxide nanosheets.


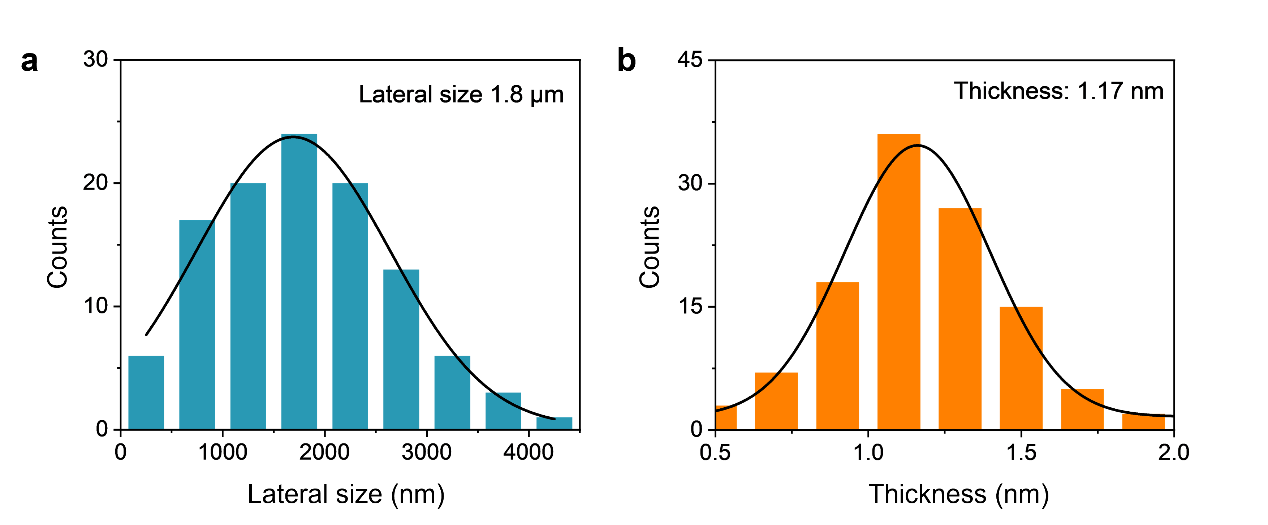


**Fig. S6** Statistics for the 2D cobalt-doped titanium oxide nanosheets’ lateral size (a) and thickness(b).

**
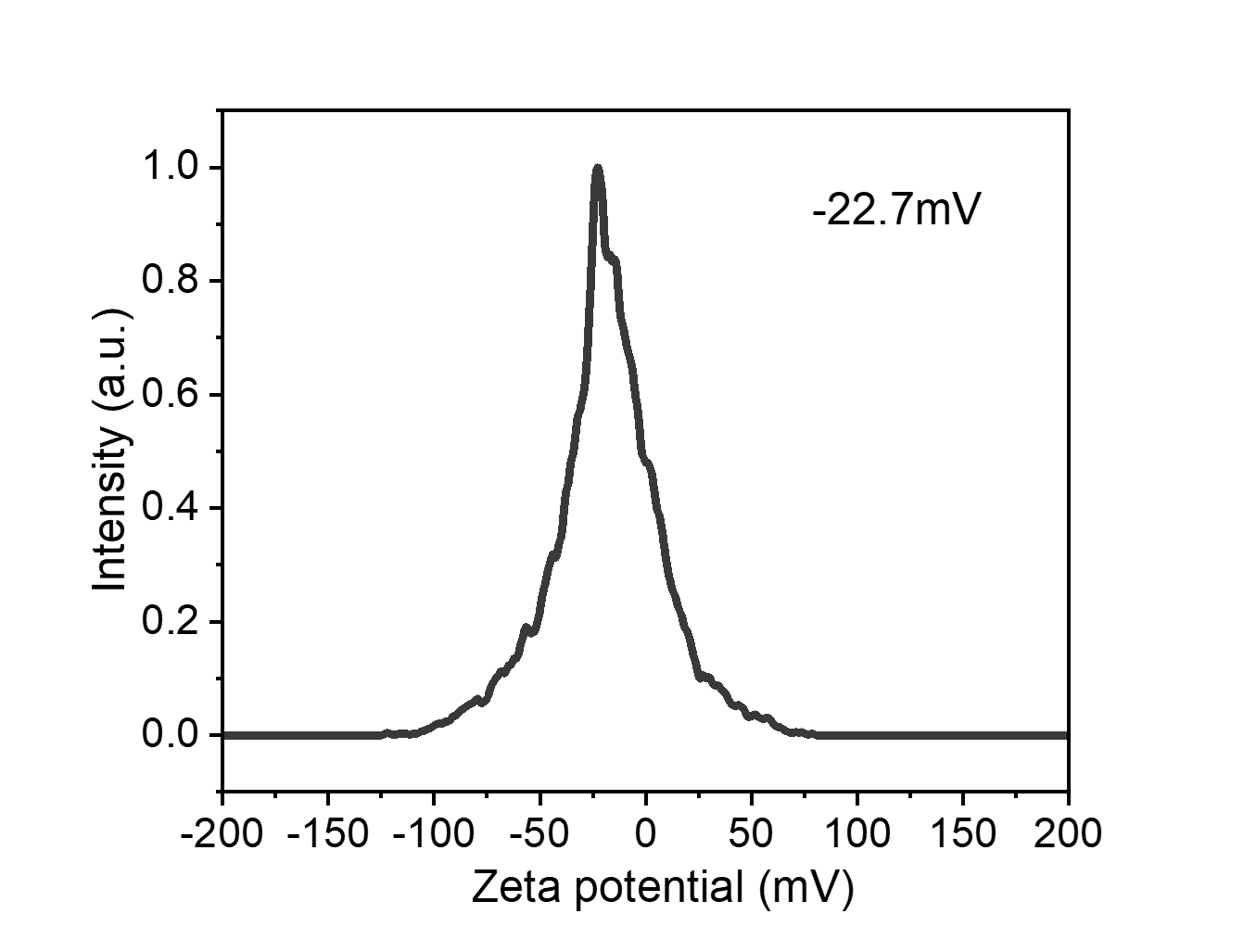
**

**Fig. S7** The zeta potential of the synthesized carbon dots/Co-TiO_2_ composites.


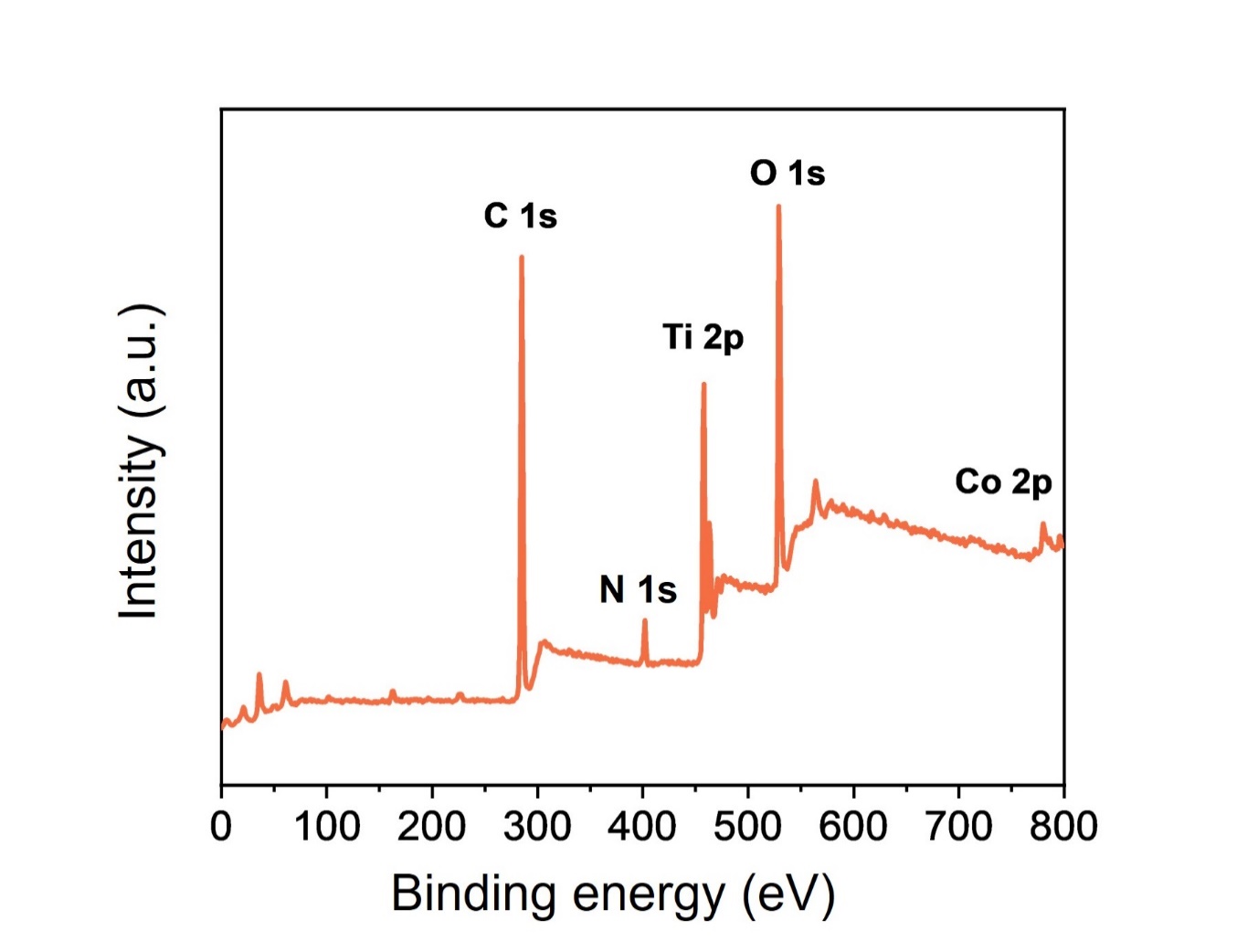


**Fig. S8** XPS spectra of Carbon dots/2D cobalt doped titanium dioxide nanosheets.


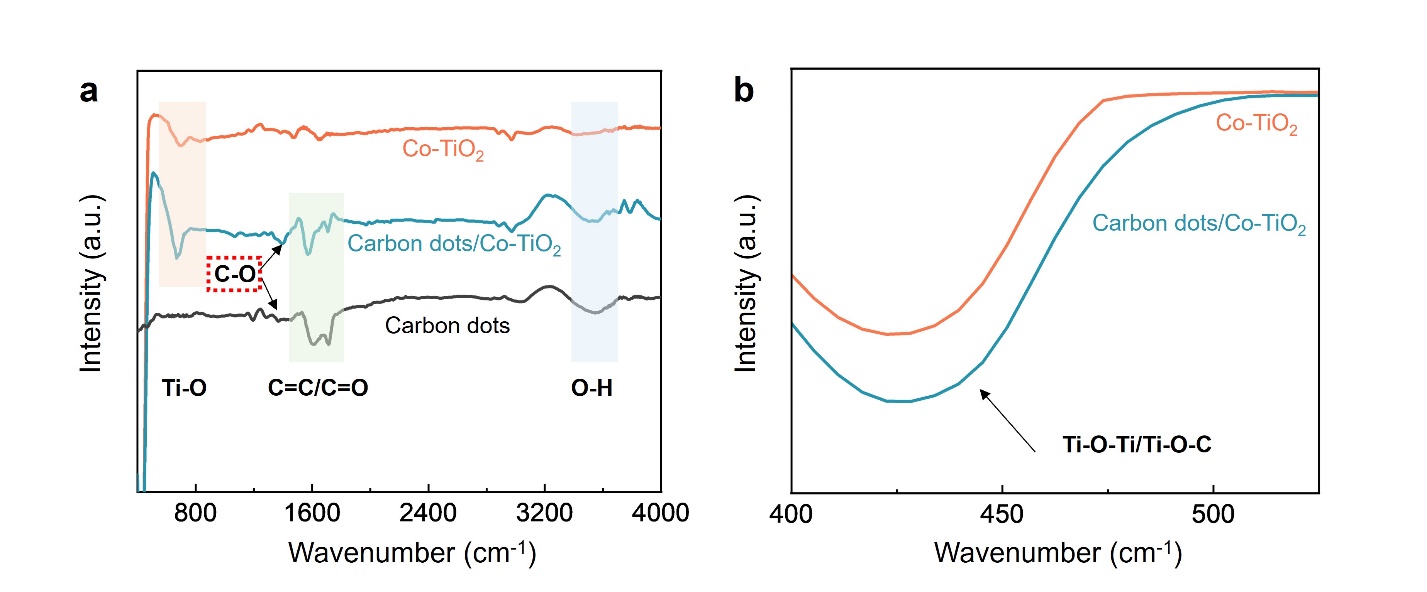


**Fig. S9** FT-IR spectra of cobalt-doped titanium oxide, the CQDs/2D cobalt-doped titanium oxide heterojunction, and carbon dots.


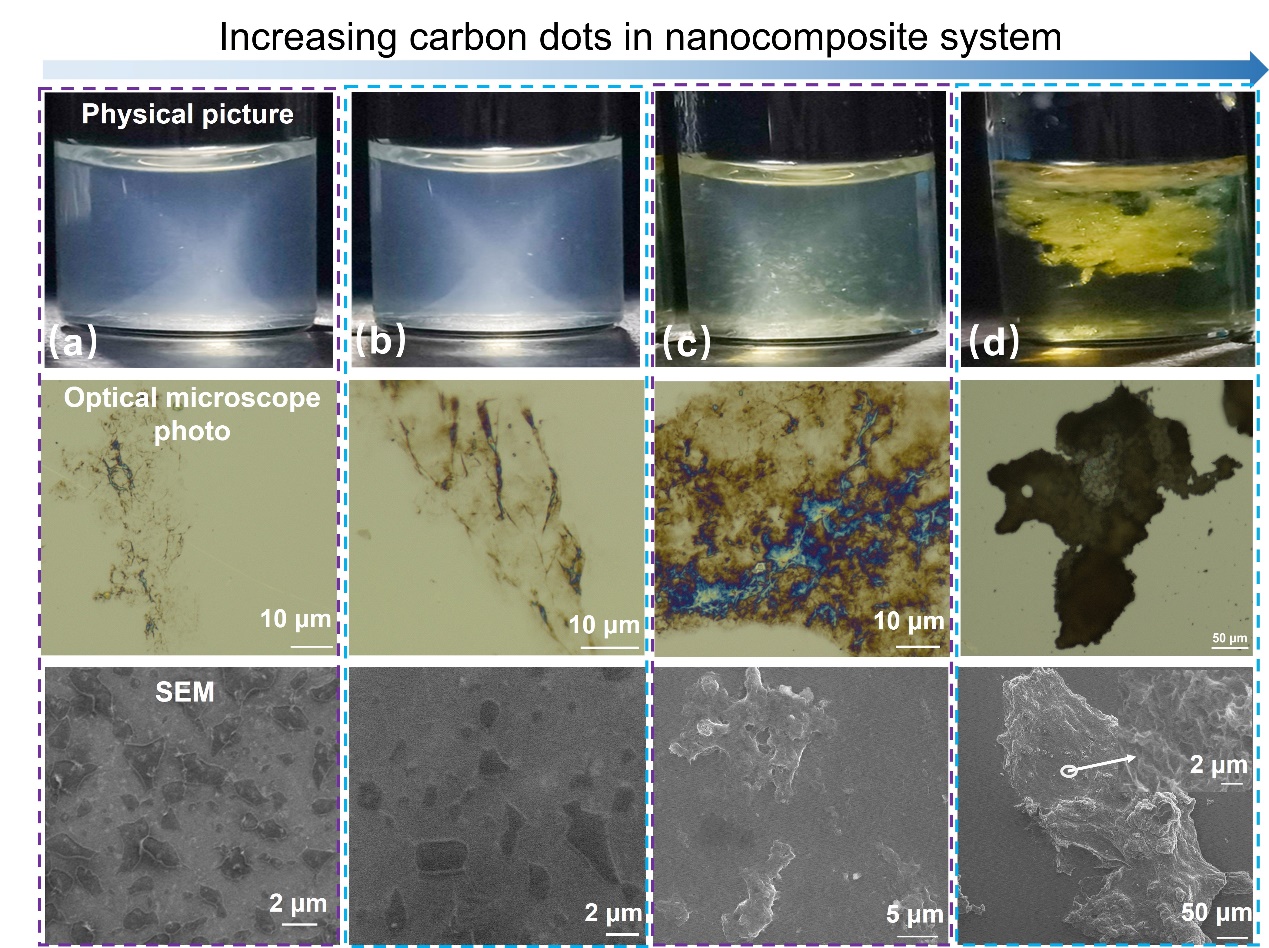


**Fig. S10** Morphology evolution of heterojunction with 1 mg/mL cobalt doped titanium dioxide nanosheets and different carbon dot concentrations: 0.025 vol‰ (a), 0.05 vol‰ (b), 0.3 vol‰ (c) and 2 vol‰ (d). (From top to bottom are Physical picture, Optical microscope photo and SEM, respectively)


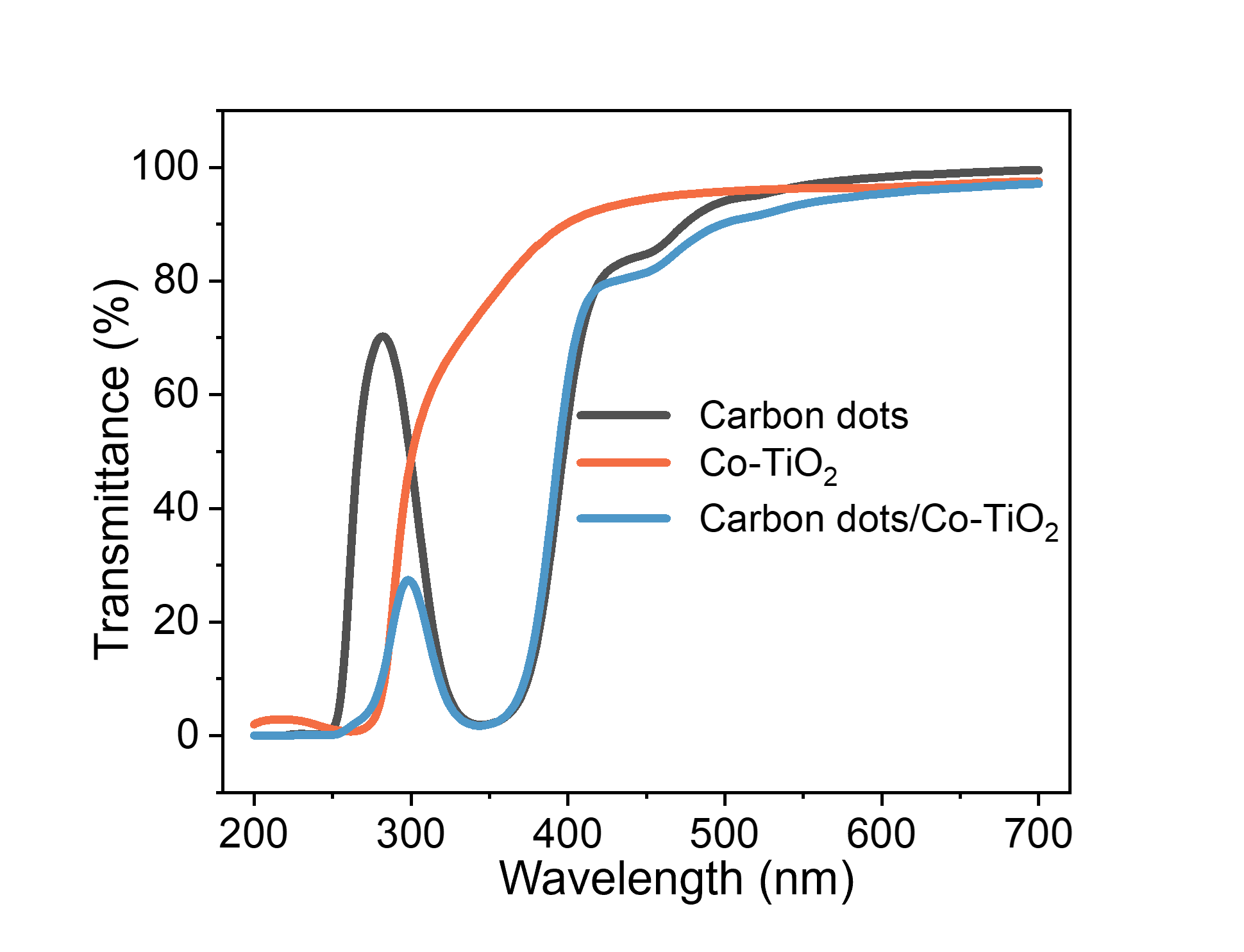


**Fig. S11** The transmittance spectrum of carbon dots, Co-TiO_2_ as well as carbon dots/ Co-TiO_2_ composites.


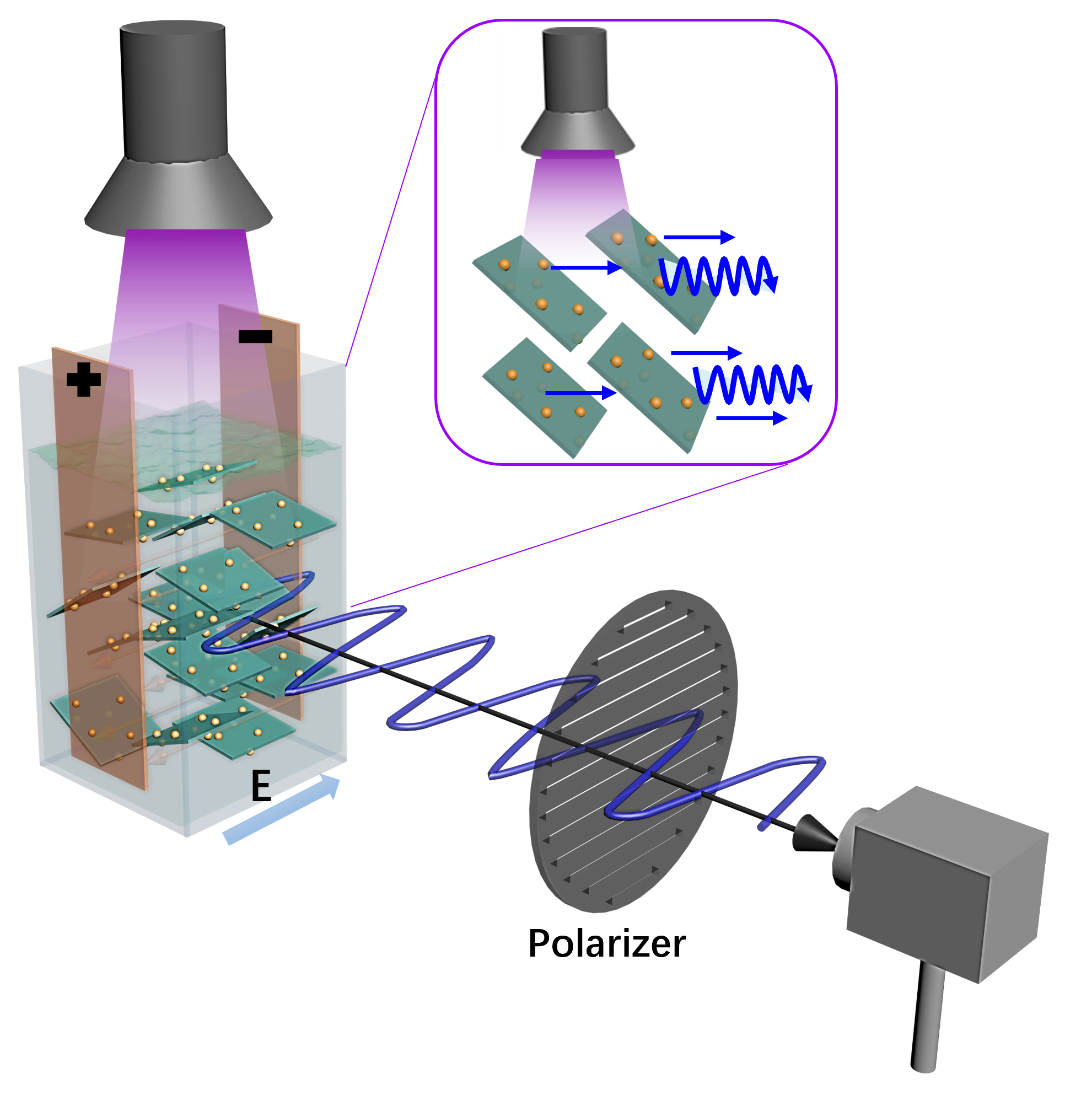


**Fig. S12** Schematic diagram of the dimming experiment principle of the fabricated device.


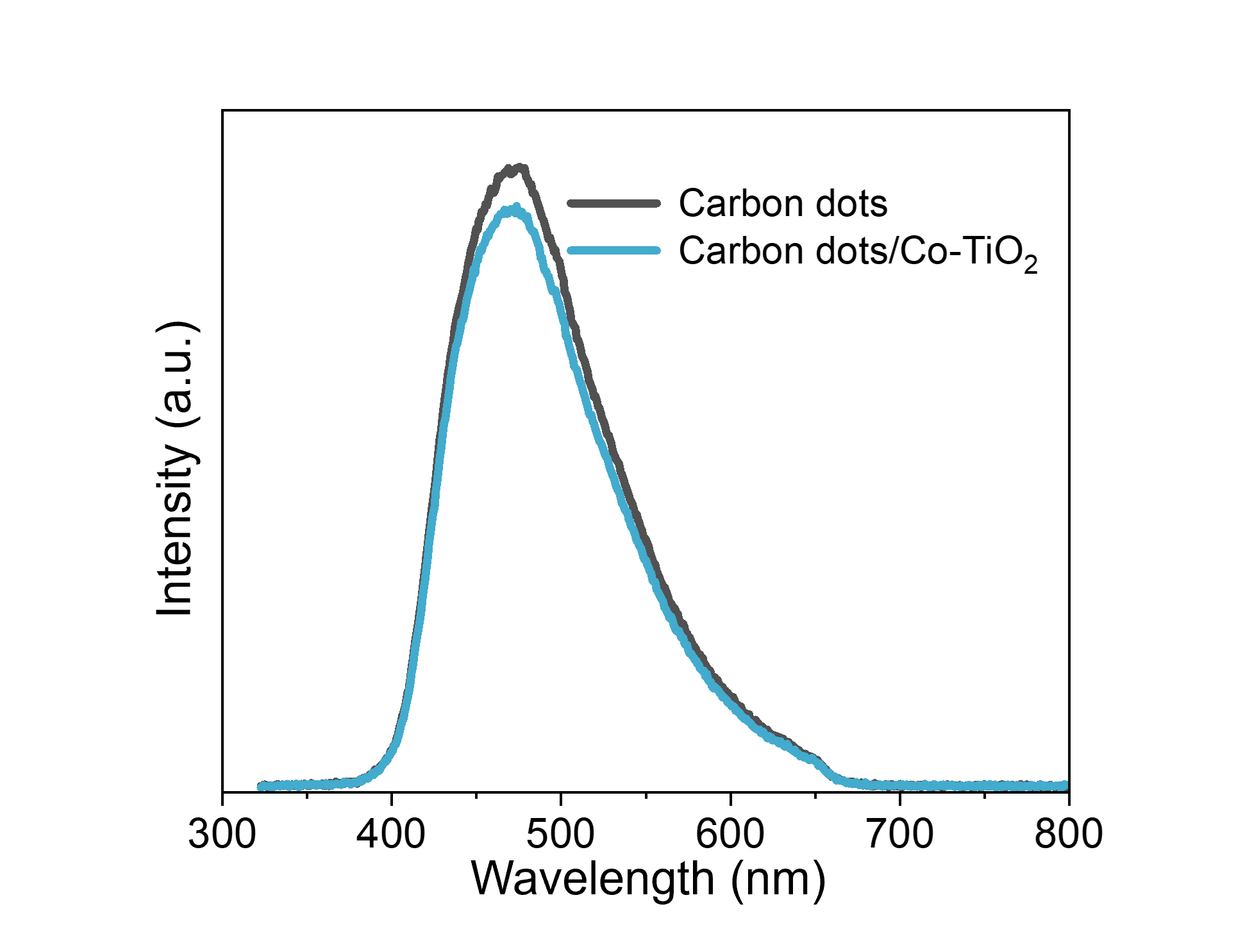


**Fig. S13** The emission spectrum of carbon dots and carbon dots/ Co-TiO_2_ composites.
